# An Observational Study of the Secondary Effects of a Local Smoke-Free Ordinance

**Published:** 2011-06-15

**Authors:** Amy A. Williamson, Brion J. Fox, Paul D. Creswell, Xiaodong Kuang, Patrick L. Remington, Sudakshina L. Ceglarek, Aaron M. Brower

**Affiliations:** University of Wisconsin Carbone Cancer Center; University of Wisconsin Carbone Cancer Center, School of Medicine and Public Health, Madison, Wisconsin; University of Wisconsin Carbone Cancer Center, School of Medicine and Public Health, Madison, Wisconsin; University of Wisconsin Carbone Cancer Center, School of Medicine and Public Health, Madison, Wisconsin; University of Wisconsin Carbone Cancer Center, School of Medicine and Public Health, Madison, Wisconsin; School of Social Work, University of Wisconsin, Madison, Wisconsin; School of Social Work, University of Wisconsin, Madison, Wisconsin

## Abstract

**Introduction:**

The secondary, sometimes unintended effects of smoke-free ordinances have not been thoroughly evaluated. In this observational study, we evaluated the association of a local ordinance implemented in Madison, Wisconsin, with changes in public disturbances; smoking, drinking, and bar-going behaviors in the general population; and smoking and drinking behaviors among university students.

**Methods:**

We obtained data from 4 sources: police records, key informant interviews, a community survey, and an undergraduate survey. Except for interviews, which we conducted postenactment only, we compared measures before and after the ordinance was put into effect.

**Results:**

We found no evidence of association of the ordinance with public disturbances. We found that the ordinance was not associated with changes in smoking rates, drinking rates, or bar-going in the general population, although bar-going decreased among the 16% of the general adult population who smokes (from 84% in 2005 to 70% in 2007, *P* < .001). Student smoking rates also decreased (from 23% in 2005 to 16% in 2007, *P* < .001), but student binge drinking did not change.

**Conclusion:**

The study adds unique information to the evidence base on the effect of smoke-free policies, finding little evidence of their secondary, unintended effects. With the addition of these results to existing evidence, we conclude that the potential health benefits of smoke-free ordinances outweigh the potential harms from unintended effects.

## Introduction

To protect the health of nonsmokers and workers from harms due to secondhand smoke, many local and state governments have enacted ordinances requiring workplaces to be smoke-free (1). Madison, Wisconsin, state capital and home of the flagship campus of the University of Wisconsin, implemented a citywide smoke-free ordinance covering virtually all workplaces, including bars and restaurants, in July 2005. Madison's previous ordinance excluded bars, restaurants with a full-service bar accounting for more than 50% of sales, bowling alleys, and restaurants with separately ventilated smoking areas.

Because of the long-standing local culture of alcohol use, including high rates of binge drinking (2), some community members were concerned that there would be secondary harms from the ordinance. These included speculation about an increase in unregulated student "house parties" and increased noise, violence, and other disturbances near bars as a result of patrons smoking outside (3). Opponents of the ordinance (eg, bar owners) raised concerns about the loss of business because Madison is surrounded by communities that did not restrict workplace smoking.

Although the health and economic impact of smoke-free ordinances has been extensively studied (4,5), there are few published studies on secondary effects of smoking bans. Other than a single study of the impact of smoking bans on driving under the influence of alcohol (6), we are unaware of any published studies of their effect on public disturbances. Despite evidence that smoking is highly correlated with alcohol consumption (7), analyses of the effect of smoke-free ordinances on community drinking rates have been few (8-10). Finally, there are no published studies of the effect of ordinances on smoking and drinking behavior in US college student populations.

The primary objective of this study was to evaluate whether the enactment of the Madison ordinance was associated with any change in public disturbances, such as noise, house parties, or other crime related to high-risk drinking. Secondary objectives were to identify whether the enactment of the ordinance was associated with changes in smoking, drinking, or bar-going behaviors and attitudes among Madison adults and in smoking and drinking behavior among university students.

## Methods

This study evaluates the effect of the Madison smoke-free ordinance by using a pre-ordinance/postordinance design based on multiple data sources: police records, key informant interviews, a community survey, and a student survey. Institutional review boards of the University of Wisconsin-Madison approved the study.

### Police records

Project staff collected incident reports from the City of Madison and the University of Wisconsin-Madison police departments. Police do not identify infractions as being alcohol-related. Instead, project staff collected the census of all incidents in 10 categories that recent research associates with high-risk drinking (that which puts self and others at risk) in Madison and at universities generally (11). The categories include noise complaints, disturbance calls, battery, and intoxicated driving. The data were compared for the 12-month period before enactment of the ordinance (July 1, 2004-June 30, 2005) and the 12-month period after (July 1, 2005-June 30, 2006) to explore the association, if any, between the smoke-free ordinance and infractions related to high-risk drinking. We compare the number of infractions as measures of true prevalence, not as estimates compared by using statistical tests.

### Key informant interviews

From April through October 2006, approximately 1 year after the ordinance was enacted, study staff conducted semistructured interviews with a purposive sample of key informant community members. The goal of the interview was to assess awareness of any change in public disturbances resulting from drinking that may be associated with the ordinance. These include house parties or large gatherings of students in off-campus locations where alcohol is served. The study team developed interview questions on the basis of pilot data, popular press reports, and literature review.

Potential informants included people whose professional duties put them in contact with the smoking ordinance and its community-level outcomes. Staff identified 44 potential participants, of whom 27 agreed to participate (a 61% response rate). For the 7 bar owners interviewed, study staff used city licensing records to identify a random sample of Madison bars stratified by location and availability of food service. For the 20 non−bar owners interviewed, study staff identified informants by contacting leaders of 19 local organizations that the investigators had reason to believe had staff meeting the inclusion criteria. These respondents represented university administration (6), government (5), police (4), business- and health-related coalitions (3), property owners (1), and providers of substance abuse health care (1).

A trained study staff member conducted the 1-hour interviews and analyzed the content of transcripts for themes related to key interview questions. The analysis involved identifying and categorizing main themes and patterns (12), following the key questions of the interview instrument. The researcher then coded interviews by theme. An investigator independently read transcripts of a subset of interviews to assess agreement with the themes and codes applied by the researcher. We resolved discrepancies through discussion of each case.

### Community survey

In March through May 2005, a few months before the ordinance was enacted, we surveyed by telephone 652 Madison adults regarding drinking and smoking behaviors, bar-going behaviors, bar preferences based on smoking rules, attitudes toward bar smoking policies, and perceptions of alcohol-related public disturbances. The sample was randomly generated from telephone numbers pertaining to all Madison zip codes, which approximates the city with 95% coverage. We calculated sample size needs on the basis of Wisconsin Behavioral Risk Factor Survey (WIBRFS) data (13). People were excluded from the survey if their household was not in Madison or if they were younger than 21 years. The respondent selection procedure was the same as that used by the Behavioral Risk Factor Surveillance System (BRFSS), except that the BRFSS minimum age is 18. The Council of American Survey Research Organizations (CASRO) response rate was 56%, similar to response rates for comparable health behavior telephone surveys (14). The survey used standard questions from the BRFSS (15) and questions developed specifically for the study.

Two years later, in March through May 2007, we administered the same survey to a new sample of 650 residents, using the same sampling methods as in 2005. The CASRO response rate to the survey was 48%. The 2005 and 2007 survey samples were statistically comparable in terms of sex, education, income, and race, although on average the 2005 survey respondents were slightly younger, fewer were married, and more were employed.

We used SPSS 13.0 (SPSS, Inc, Chicago, Illinois) to analyze the community and university survey data. We used the 2 proportion* z* test to compare differences in proportions and chose *P* < .05 as the level of significance.

### Survey of university undergraduate students

To assess University of Wisconsin-Madison undergraduate students' smoking and drinking behavior pre-ordinance and postordinance, we analyzed data from the 2005 and 2007 University of Wisconsin System Alcohol and Other Drug Use Survey (UWS-AODA Survey) (Board of Regents of the University of Wisconsin System, unpublished report). The UWS-AODA Survey incorporates items from the Core Alcohol and Drug Survey, which has been tested for reliability, consistency, and validity (16).

The University of Wisconsin-Madison administered the UWS-AODA Survey via e-mail to a random sample of all undergraduates enrolled as of January 2005 (n = 1,503) and January 2007 (n = 2,000). A total of 536 students completed the survey in March 2005 (a 36% response rate), and 1,008 students responded in March 2007 (a 50% response rate). These response rates are comparable with those of other behavioral surveys implemented among University of Wisconsin-Madison students (17). Survey respondents in 2005 and 2007 were similar in terms of sex, race, and student status. Postordinance, the sample was slightly younger (Student's *t* test, *P* = .01) and more respondents lived off campus (χ^2^ test, *P* < .05). In both years, post hoc sample balancing was used to adjust the sample to better represent the sampling frame in terms of sex distribution. To analyze the survey results, we used the 2 proportion *z* test to compare differences in proportions (*P* < .05 level of statistical significance).

## Results

### Public disturbances

According to city police records, most incidents related to high-risk drinking decreased during the postordinance year (Table 1). Compared with the pre-ordinance year, calls related to fights, intoxicated persons, and trespassing declined by more than 5% during the postordinance year. Noise complaints and vandalism also decreased slightly. However, liquor law violations (including fake identification cards and selling to a minor) increased by 15%. Other data sources did not provide an interpretation of this result. The police records also revealed slight increases in battery and disturbance calls.

Police data reveal that during the postordinance year, incidences of intoxicated driving in the city were down by 2% (Table 1). Results from the university student survey showed that postordinance, significantly fewer students reported operating a car under the influence of alcohol (24% in 2005 vs 18% in 2007, 2 proportion* z *test, *P* = .005) or riding in car with an intoxicated driver (10% vs 7%, 2 proportion* z *test, *P* = .05) (Table 2).

The community survey found that fewer respondents observed alcohol-related public disturbances in 2007 than in 2005 (9% vs 12%, 2 proportion* z *test, *P* = .04) (Table 3). Similarly, key informant interviews found little evidence of change in drinking-related public disturbances. All informants agreed that the number of people lingering outside of bar entrances to smoke had increased postordinance, but there was no consensus on whether this had led to disturbances. One-third of all participants believed that it had led to an increase in disturbances, whereas two-thirds either thought it had no effect or did not know. Those who believed that the lingering had not led to disturbances included people with closest knowledge of the problem, such as police officers, some bar owners, and a city employee with related professional responsibility. All but 1 of the 4 police officers interviewed noted no increase in calls for service associated with people lingering outside of bars. A higher proportion of bar owners than other participants believed that lingering had led to an increase in disturbances.

The key informants were also asked whether they were aware of any effect of the ordinance on the number, size, or disruptive nature of university student house parties. Nearly all of the 27 key informants either asserted that the smoking ordinance had not had an effect on house parties (n = 12) or that they  did not know (n = 14). All of those with professional experience directly related to house parties (university administrators and property owners) were certain that the smoking ordinance had not had an effect on these parties.

### Community resident smoking and drinking behaviors and attitudes

The results of the community survey indicate that no significant changes in smoking rates and quit rates occurred from 1 year pre-ordinance through 2 years postordinance among adult Madison residents (Table 3). Nearly 16% of respondents were smokers in both years. About 70% of respondents reported having had a drink during the past 30 days in both 2005 and 2007, and the binge drinking rate was nearly 24% in both years. Although the drinking rate decreased among smokers from 76% in 2005 to 67% in 2007, the difference was not significant.

The same survey found modest changes in bar-going behavior between 1 year pre-ordinance and 2 years postordinance (Table 3). In both years, approximately 70% of all respondents reported going to bars. However, among smokers postordinance, bar-going decreased significantly (from 84% in 2005 to 70% in 2007 [2 proportion* z *test, *P* < .001]). The survey also found that fewer bar patrons reported going to bars inside the Madison city limits in 2007 (74%) compared with 2005 (84%, 2 proportion* z *test, *P* < .001), whereas in 2007 more bar patrons reported going to bars outside Madison in the surrounding county.

Residents expressed greater preference for nonsmoking bars and stronger support for smoke-free policy in the postordinance year. Between 2005 and 2007, the proportion of respondents reporting a preference for smoke-free bars when making a decision to go out increased significantly (46% vs 54%, 2 proportion* z *test,* P* = .002). This was true for both smokers and nonsmokers. Also between 2005 and 2007, the proportion of respondents who believed that smoking should not be allowed in bars increased significantly. This was true for all respondents (40% vs 47%, 2 proportion* z *test, *P* = .007) and for nonsmokers (45% vs 54%, 2 proportion* z *test, *P* = .001). The increase in the proportion of smokers who believed smoking should not be allowed (9% vs 11%) was not significant.

### University student smoking and drinking behaviors

The university student survey showed a significant drop in the smoking rate among students during the 2 years after enactment of the ordinance;16% of respondents used cigarettes at some point in the past 30 days in 2007, compared with 23% in 2005 (2 proportion* z *test, *P* < .001) (Table 2).

In contrast, alcohol use varied only slightly between the 2 surveys (Table 2). In both years, nearly 85% of respondents indicated that they had used alcohol at some point in the past 30 days. However, although overall binge drinking was unchanged (at nearly two-thirds of students), the rate of frequent binge drinking (defined as 3 or more times in the past 2 weeks) declined between 2005 and 2007 (36% vs 30%, 2 proportion* z *test, *P* = .006).

## Discussion

Using multiple data sources, we found little evidence during a 1- to 2-year period after enactment of the Madison smoke-free ordinance of increased public disturbances, such as house parties, intoxicated driving, or disruption caused by smokers lingering outside of bars. These findings run counter to concerns raised by ordinance opponents. That alcohol-related crime did not increase postordinance is particularly relevant because violent crime in the downtown area increased during the same period (18).

Our results differ from those of the only other published study that has addressed the effect of smoking bans on intoxicated driving. In a multisite study that did not include Wisconsin, Adams and Cotti (6) observed an increase in fatal accidents involving alcohol in counties with smoking bans in place. In our study, we did not analyze intoxicated driving incidents at the county level, although advocacy organizations reported a nearly 9% decrease in injuries due to alcohol-related crashes in Dane County, in which Madison lies, during 2005 through 2007 (19).

Our study findings suggest that the smoke-free ordinance was not associated with a change in smoking rates in the general population. (Wisconsin cigarette taxes did not change during the study period.) Although smoke-free ordinances can reduce smoking rates (20,21), these changes may take more time to observe (22). Indeed, our finding of postordinance increases in support for smoke-free policy and preferences for smoke-free establishments points to a change in the community's views on smoking, which can indirectly reduce smoking prevalence (23).

The smoking rate among university students dropped significantly in the 2 years postordinance. The decrease of 7percentage points may be explained by underlying trends in smoking in this demographic cohort. In Wisconsin, data from the WIBRFS (the only data against which to test this claim) showed a decrease of 6 percentage points (from 34% to 28%) in smoking among youth aged 18 to 29 years during the same period, 2005 through 2007 (13). Because little research has been published on risk factors for smoking among college students versus nonstudents in this age cohort, comparison of these WIBRFS data with our study requires a degree of caution. Because most university students live downtown or on campus and lack access to cars, they are inclined to frequent bars within the city limits. Many university students are in the initiation phases of smoking (24). It stands to reason, therefore, that if students experience fewer opportunities to pair smoking with alcohol consumption — a behavior correlated with smoking initiation (25) — they may be less likely to initiate smoking at all. This association should be studied further.

As noted above, few studies have examined the effect of smoke-free ordinances on drinking behavior at the local level, especially within the United States. Our study did not find significant changes in drinking rates in the general adult population but did observe a reduction in frequent binge drinking among university students. The reduction in frequent binge drinking may be due to factors unrelated to the ordinance; similar results were observed at other University of Wisconsin campuses across the state (26).

Results were mixed regarding association of the ordinance with changes in bar-going, including evidence of a decrease in bar-going among smokers in Madison and an increase in bar patrons frequenting bars outside of Madison (where smoking was still permitted). More extensive objective data (eg, sales and tax receipts, employment statistics) would be necessary to address the effect on bar business definitively (4). The City of Madison and others reported an increase in establishments with liquor licenses in the 2 years postordinance (27,28). These reports are not necessarily incompatible with our findings, but they indicate that the financial effect of changes in bar-going behavior may be small overall. The mixed results on bar-going behavior should be considered in the context of the results for university students. In Madison, students as a group are less able to change their drinking location in response to the ordinance. The reduction in student smoking rates points to the potential individual and public health benefits of statewide smoking bans, since most residents will not drive to a different state to drink and smoke.

There are several limitations of this study. First, the study design does not permit clear determination of causation in the changes observed. To increase the validity of the results, future research should include several years of data before and, ideally, after implementation of similar ordinances, and should use statistical methods that control for secular trends and random effects (4). In the absence of more extensive data, we sought to increase the relevance of our results by comparing them with statewide trends during the same period. Statewide drinking and smoking rates observed in the WIBRFS (13) and the University of Wisconsin System AODA Survey (26) were similar to those from our study, which supports the interpretation that the ordinance did not affect these behaviors. There are no statewide data on the alcohol-related crimes examined in this report. The most comparable data, statewide property crimes, increased 4% between 2005 and 2007 (29). Thus, the decrease in alcohol-related incidents we observed was not likely part of a downward secular trend in related crimes.

Second, our evaluation of the economic impact of the ordinance was limited to assessment of the bar-going preferences of survey respondents. As previously noted, analysis of more extensive objective data is required for definitive evidence (4). Third, we were unable to examine intoxicated driving incidents at the county level. Further analysis is required to test whether there were more intoxicated driving incidents outside the city caused by bar patrons driving to neighboring communities (6).

Although the study was not designed to provide generalizable results, it adds unique information to the evidence base on the impact of smoke-free policies. Contrary to claims of ordinance opponents, we found little to no evidence of a rise in public disturbances, no increase in binge drinking rates, and a notable decrease in student smoking rates. Although some change in bar-going behavior was observed, we found that the ordinance was not associated with changes in drinking rates in the general population. With the addition of these results to existing knowledge, we conclude that the potential health benefits of smoke-free ordinances far outweigh the potential harms.

## Figures and Tables

**Table 1:** Police Incidents Related to High-Risk Drinking^a^ in the City of Madison, Wisconsin: 1 Year Pre-Ordinance Compared With 1 Year Postordinance^b ^

**Incident type**	No. of Incidents Pre-Ordinance^c^	No. of Incidents Postordinance^c^	Percentage Change Between Pre-Ordinance and Postordinance Period^d^
Battery^e^	2,009	2,076	3
Disturbance calls	4,618	4,730	2
Fight calls	941	861	−9
Intoxicated driving	704	690	−2
Intoxicated person	520	475	−9
Liquor law violations	1,210	1,387	15
Noise complaints	5,509	5,391	−2
Threats	1,244	1,254	0.8
Trespassing	942	847	−10
Vandalism	4,412	4,217	−4

Source: City of Madison and University of Wisconsin-Madison police departments, unpublished data.

a Drinking that puts self or others at risk.

b The pre-ordinance and postordinance data are the census of all such incidents occurring in Madison during these periods. As such, they represent true prevalence, not estimates compared statistically as samples.

c The pre-ordinance period is July 1, 2004-June 30, 2005; the postordinance period is July 1, 2005-June 30, 2006.

d Percentages are rounded.

e Includes aggravated battery.

**Table 2:** Rates of Smoking, Drinking, and Driving While Intoxicated Among University of Wisconsin-Madison Undergraduates, 2005 and 2007

Characteristic	2005 (n = 536), %	2007 (n = 1,008), %	*P* Value^a^
Current smoker^b^	23	16	<.001
Current drinker^c^	84	86	.17
Binge drinker^d^	65	63	.17
Frequent binge drinker^e^	36	30	.006
Drove a car under the influence of alcohol	24	18	.005
Rode in car with an intoxicated driver	10	7	.05
Arrested for driving under the influence of alcohol or driving while intoxicated	1	1	.50

Source: University of Wisconsin System Alcohol and Other Drug Use Survey for University of Wisconsin-Madison (Board of Regents of the University of Wisconsin System, unpublished report).

a Two proportion *z* test.

b Used cigarettes at some point in the past 30 days.

c Used alcohol at some point in the past 30 days.

d Consumed at least 5 drinks in 1 sitting in the past 2 weeks.

e Consumed at least 5 drinks in 1 sitting 3 or more times in the past 2 weeks.

**Table 3:** Smoking and Drinking Attitudes, Behaviors, and Observations of Adult Residents of Madison, Wisconsin, 2005 and 2007

**Characteristic**	2005 (n = 652), %	2007 (n = 650), %	*P*Value^a^
**Current smoker^b^**	16	16	.76
**Stopped smoking 1 day or longer in the past 12 months because were trying to quit (among current smokers)**	50	47	.56
**Current drinker^c^**			
All survey respondents	70	68	.46
Smokers	76	67	.08
Nonsmokers	68	69	.93
**Binge drinker^d^ (among drinkers)**	25	23	.43
**Report going to bars**			
All survey respondents	69	66	.15
Smokers	84	70	<.001
Nonsmokers	66	65	.38
**Of bar patrons, usually frequent bars in Madison**	84	74	<.001
**Of bar patrons, usually frequent bars in Dane County but outside Madison**	12	20	<.001
**Report preference for smoke-free bars**			
All survey respondents	46	54	.002
Smokers	6	17	<.001
Nonsmokers	53	61	.002
**Believe that smoking should not be allowed in bars**			
All survey respondents	40	47	.007
Smokers	9	12	.10
Nonsmokers	45	54	.001
**Observed alcohol-related incidents in public^e^**	12	9	.04

a Two proportion *z* test.

b Have smoked at least 100 cigarettes in their lifetime and currently are smoking some days or every day.

c Had a drink in the past 30 days.

d Had 5 or more drinks on 1 occasion in the past 30 days.

e Such as car accident, violence, street noise or disturbances, fighting, public urination, or property damage.
